# High Levels of Sequence Diversity in the 5′ UTRs of Human-Specific L1 Elements

**DOI:** 10.1155/2012/129416

**Published:** 2012-02-07

**Authors:** Jungnam Lee, Seyoung Mun, Thomas J. Meyer, Kyudong Han

**Affiliations:** ^1^Department of Nanobiomedical Science & WCU Research Center, Dankook University, Cheonan 330-714, Republic of Korea; ^2^Department of Microbiology, College of Advance Science, Dankook University, Cheonan 330-714, Republic of Korea; ^3^Department of Biological Sciences, Louisiana State University, Baton Rouge, LA 70803, USA

## Abstract

Approximately 80 long interspersed element (LINE-1 or L1) copies are able to retrotranspose actively in the human genome, and these are termed retrotransposition-competent L1s. The 5′ untranslated region (UTR) of the human-specific L1 contains an internal promoter and several transcription factor binding sites. To better understand the effect of the L1 5′ UTR on the evolution of human-specific L1s, we examined this population of elements, focusing on the sequence diversity and accumulated substitutions within their 5′ UTRs. Using network analysis, we estimated the age of each L1 component (the 5′ UTR, ORF1, ORF2, and 3′ UTR). Through the comparison of the L1 components based on their estimated ages, we found that the 5′ UTR of human-specific L1s accumulates mutations at a faster rate than the other components. To further investigate the L1 5′ UTR, we examined the substitution frequency per nucleotide position among them. The results showed that the L1 5′ UTRs shared relatively conserved transcription factor binding sites, despite their high sequence diversity. Thus, we suggest that the high level of sequence diversity in the 5′ UTRs could be one of the factors controlling the number of retrotransposition-competent L1s in the human genome during the evolutionary battle between L1s and their host genomes.

## 1. Introduction

Transposable elements are a considerable component of the human genome, responsible for approximately 45% of the human genome sequence [1]. These elements are associated with genomic instability via *de novo* insertions, insertion-mediated deletions, and recombination events [2–8] and are responsible for a number of genetic disorders [9]. Almost all of the transposable elements belong to one of four types: long interspersed elements (LINEs), short interspersed elements (SINEs), long terminal repeat (LTR) retrotransposons, and DNA transposons [1, 10–12]. Among them, LINE-1s or L1s are one of the most successful retrotransposon families in the human genome, with 516,000 copies comprising 17% of the human genomic sequence [1]. A full-length functional L1 element is about 6 kb in length and contains a 5′ untranslated region (UTR) bearing an internal RNA polymerase II promoter, two open reading frames (ORF1 and ORF2), and a 3′ UTR terminating in a poly(A) tail [13]; ORF1 encodes an RNA-binding protein that has demonstrated nucleic acid chaperone activity *in vitro*, and ORF2 encodes a protein with both endonuclease (EN) and reverse transcriptase (RT) activities, which are required for L1 retrotransposition [14–16]. The generally accepted model for L1 retrotransposition is called target-primed reverse transcription (TPRT). In this mechanism, the L1 RNA forms a ribonucleoprotein complex with its own encoded proteins and then moves back to the nucleus where the L1 EN makes a single-stranded nick producing a free 3′-hydroxyl at the end of a poly(T) overhang to which the 3′ poly(A) tail of the L1 RNA anneals. The L1 RT then primes at this site and initiates reverse transcription. In addition to the newly inserted L1 sequence, TPRT typically generates 7-to-20 bp target site duplications flanking each side of the L1 insertion [17, 18].

Most previous studies of human-specific L1s focused on the 3′ UTRs rather than the 5′ UTRs because over 90% of L1 elements are 5′ truncated [19, 20], a result of the termination of reverse transcription, for unknown reasons, before the synthesis of a new L1 copy is complete. The 5′ UTR-deficient L1 is unable to propagate because it does not contain a promoter as well as transcription factor (TF) binding sites such as Yin Yang 1 (YY1), two putative SRY-related TF binding sites, and two putative RUNX3 TF binding sites [21]. These truncated insertions are therefore unlikely to influence the evolutionary history of L1 elements. It has been reported that several distinct lineages of L1s propagated in primate genomes simultaneously during the first 30 million years (myrs) of primate evolution, between 70 and 40 myrs ago. However, only a single lineage of L1 elements appears to have been retrotranspositionally active over the last 40 myrs. Although competition between distinct L1 lineages for host factors has been suggested as a reason why a single lineage came to dominance in the human genome for the last 40 myrs, a comprehensive explanation remains unclear [22].

The human genome contains ~2,000 copies of human-specific L1s [23, 24]. However, among them, only approximately 80 L1 copies are thought to be able to retrotranspose actively in the human genome. These are called retrotransposition-competent L1s and may be further divided into two groups, the “active” and “dead” L1s, according to whether they can be shown to retrotranspose *in vitro* [25]. To explore any connection between the rate of substitution and the regulatory elements residing within the L1 5′ UTR, we identified substitutions along the 5′ UTR sequences of the human-specific L1s. In addition, we estimated the mutation rate of each component of the human-specific L1s, based on the divergence among them. The comparison of the mutation rate of the L1 5′ UTRs with those of other L1 structural components (ORF1, ORF2, a partial of ORF2 and 3′ UTR, and the 3′ UTR) indicates that the 5′ UTR has accumulated mutations at a faster rate than the other components. Furthermore, an analysis of the CpG sites in all components found no correlation between the CpG contents and mutation rates of the L1 components.

## 2. Materials and Methods

### 2.1. Dataset

A total of 1,835 human-specific L1 elements were previously identified [23]. We manually inspected them to extract full-length L1 elements because this study focuses on the L1 5′ UTR. This manual inspection process yielded 443 full-length L1 elements including 897 bp of the 5′ UTR sequence. We had previously identified the genomic positions of the 443 human-specific L1 elements in hg17 (UCSC May 2004 freeze) of the human genome reference sequence [23]. We converted these hg17 positions into positions within the current hg19 (UCSC February 2009 freeze) assembly of the human genome reference sequence using the BLAST-Like Alignment Tool (BLAT) utility (http://genome.ucsc.edu/cgi-bin/hgBlat) [26]. The genomic loci of the set of full-length human-specific L1s are described in Table 1 in supplementary material available online at doi: 10.1155/2012/129416.

### 2.2. Estimation of Substitution Frequencies on L1 5′ UTRs

In a previous study of human-specific L1s, six L1 subfamilies were established based on the diagnostic mutations that are shared between all members of each subfamily [23]. In this study, we grouped the 443 full-length L1 elements into the six subfamilies, L1Hs-Ta1, L1Hs-Ta0, L1Hs-preTa, L1Hs-1AB, L1PA2, and L1PA3, based on the previously established taxonomy [23]. However, the previous study had examined only a partial segment of each L1 element, an 864 bp segment corresponding to the 3′ end of ORF2 and the entire 3′ UTR. It was therefore necessary for us to construct a full-length consensus sequence for each of the L1 subfamilies. Using the module MegAlign, available in the package DNAStar, we generated the consensus sequence for each L1 subfamily.

For the subfamily of L1Hs-preTa, we aligned the 5′ UTRs of all members and compared them with its consensus sequence using the biological sequence alignment editor BioEdit v.7.0 [27]. For the alignment, we discarded the first nine base pairs of the 5′ UTR because the most 5′ end of human L1s is known to be highly variable [20]. In Excel, we generated a matrix of substitutions relative to the consensus sequence at each nucleotide position within the 5′ UTR throughout all elements in the alignment. Using the same method, we examined the substitutions existing within the 5′ UTRs of the other L1 subfamilies represented in our dataset. Finally, we summed the substitution numbers counted from each subfamily to calculate the substitution frequencies per nucleotide position across all human-specific L1 5′ UTRs.

### 2.3. Age Estimates of Human-Specific L1s Based on L1 Components

All of the human-specific L1 elements were divided into their 5′ UTR, ORF1, ORF2, and 3′ UTR components using a combination of the L1Xplorer (http://line1.bioapps.biozentrum.uni-wuerzburg.de/l1xplorer.php) annotation tool [28] and manual inspection. The sizes of the 5′ UTR, ORF1, ORF2, and 3′ UTR were 897, 1017, 3828, and 202 bp, respectively. We grouped the L1 components based on the categories of subfamily and component and aligned the members of each group using the BioEdit v.7.0 software [27]. The relationship of each component within an L1 subfamily was reconstructed using a median-joining network [29, 30], as implemented in the NETWORK 4.6 (http://www.fluxus-engineering.com/sharenet.htm) software [29]. Then, the ages of the human-specific L1s were estimated based on the divergence among all the copies of each group. For this calculation, we used 0.15% per site per myr as the nucleotide mutation rate [31]. A previous study of human-specific L1s suggested that this method is useful in estimating the age of human-specific L1s [23]. To compare the observed mutation rates between the L1 components, we assumed that older apparent age was indicative of a higher mutation rate for the component.

### 2.4. Analysis of the CpG Contents of the Four L1 Components

Using an in-house Perl script, we counted the numbers of CpG and GpC sites on the consensus sequence of each L1 subfamily. To compare CpG contents among the four L1 components, we calculated the ratio of CpG to GpC for each component. Pearson's correlation coefficient (*r*) between CpG contents and mutation rates of the L1 components was calculated. The two-tailed *P* value (i.e., statistical significance) was considered using the online freely available software, GraphPad QuickCalcs (http://www.graphpad.com/quickcalcs/index.cfm).

## 3. Results and Discussion

### 3.1. Substitutions within the 5′ UTRs of Full-Length Human-Specific L1s

**Figure 1:**
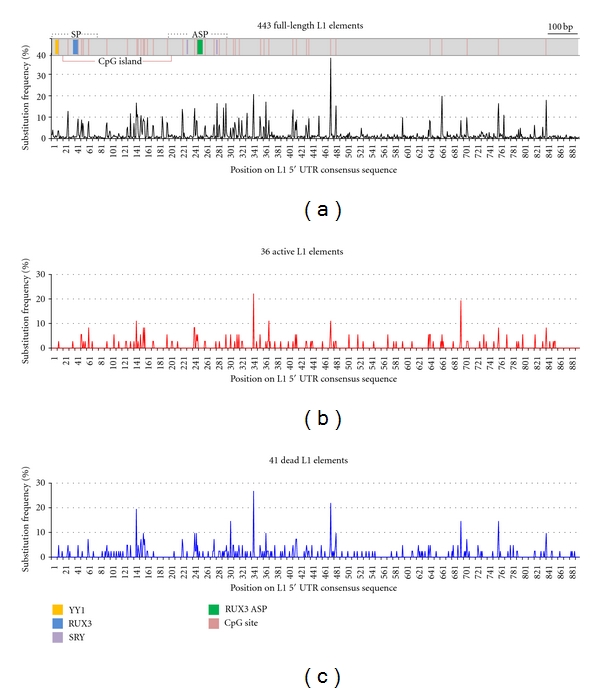
Substitution frequencies along the 5′ UTR sequences of human-specific L1 elements. The percentage of substitutions per nucleotide position along the 5′ UTR sequence was calculated. The structure of the L1 5′ UTR is shown on the top. The TF binding sites, sense promoter (SP), antisense promoter (ASP), CpG sites, and CpG islands are indicated by colored boxes and lines. (a) 443 full-length human-specific L1 elements. (b) 36 active L1 elements *in vitro.* (c) 41 dead L1 elements *in vitro*.

L1 elements are transcribed by RNA polymerase II, but an internal promoter within the 5′ UTR is independent of the TATA-box. L1 transcription is initiated at variable positions within the L1 5′ UTR. Thus, it is clear that the 5′ UTR plays an important role in regulating the initiation of L1 transcription. Sequence differences within the 5′ UTR can result in different transcriptional activities because single or a combination of nucleotide differences within the L1 5′ UTRs influences the promoter activity [32]. From a previous study of human-specific L1s [23], we identified 443 full-length L1 elements and grouped them into six different L1 subfamilies using their diagnostic substitutions. By comparing the sequences of the L1 5′ UTRs with the respective consensus sequence for each subfamily, we identified mutations within the 5′ UTRs of the 443 human-specific L1s.  Figure 1(a) shows the substitution frequency per nucleotide position among the L1 5′ UTRs. The colored boxes indicate TF binding sites: a YY1 binding site, two putative SRY-related TF binding sites, and two putative RUNX3 TF binding sites. As can be seen in Table 1, the average frequency of substitutions across the entire length of the L1 5′ UTR is 1.62%. It has been suggested that the coexistence of distinctive L1 5′ UTRs is unstable in the host genome because they compete with one another for host factor(s) which is/are required for L1 transcription. Thus, we expected that the TF binding sites would show different substitution patterns from the other regions within the L1 5′ UTR. The results of this analysis show that substitution frequencies in the TF binding sites were indeed far lower than the average frequency, except for the YY1 binding site, which showed a similar substitution frequency to the average frequency. This result makes some sense in light of the previous finding that the YY1 binding site is not required for L1 retrotransposition [21]. However, it also presents a bit of a paradox because the presence of the YY1 binding site is the only feature common to all of the seven distinctive types of L1 5′ UTR that emerged during last 70 myrs of L1 evolution [22].

 Unlike other regulatory sequences, the promoter within the L1 5′ UTR showed a higher substitution frequency than the overall average, as can be seen in Table 1. We believe that the high mutation rate of L1 5′ UTR can be largely attributed to the high mutation rate of the L1 promoter. To initiate L1 transcription, RNA polymerase II needs to recognize and bind to the L1 promoter. Thus, mutations that accumulate within the L1 promoter have the potential to reduce L1 retrotransposition activity and the number of retrotransposition-competent L1s in the human genome. We suggest that any host defense system(s) against L1 elements may be among the factors contributing to the high substitution frequency observed within the L1 promoter region of the 5′ UTR.

In one recent study of the retrotransposition activity of human-specific L1s, 82 human-specific L1s with intact ORF1 and ORF2 were identified; the ORFs encode proteins that are essential for L1 retrotransposition. In the study, the L1s were cloned to test L1 retrotransposition in cultured cells. In the cell culture assay, 40 L1s were experimentally shown to be active, while the others appear retrotranspositionally inactive in human cells [25]. Because both of the two groups retain intact ORF1 and ORF2, we reasoned that the sequence difference between their 5′ UTRs is responsible for the difference between their retrotransposition abilities. As seen in Figures 1(b) and 1(c), we examined the substitution frequency per nucleotide position on the 5′ UTRs of these two L1 groups. No significant difference between the substitution patterns of the two groups was detected: the substitution frequencies of “active” and “dead” L1 5′ UTRs averaged 0.53% and 0.78%, respectively. While at first glance this result seems surprising, we believe that it is reasonable. As mentioned previously, the initial step in L1 retrotransposition is transcription, and the 5′ UTR is known to regulate this event.* In vivo*, only the L1 elements that have a functional 5′ UTR are able to retrotranspose. In contrast, in the cell culture assay of L1 retrotransposition, it did not matter whether the L1s had a functional 5′ UTR for the initiation of L1 transcription as the transcription of both “active” and “dead” Lls was initiated from the promoters of cloning vectors. In other words, the two L1 groups do not directly represent retrotransposition-competent L1s and retrotransposition-incompetent L1s *in vivo*. Thus, it is possible that some or most of the “active” L1s previously reported have no functional 5′ UTR *in vivo*. Regarding that all of the “dead” L1s are presumably inactive *in vivo*, it was not a surprising result that the two different L1 groups have similar substitution patterns within their 5′ UTRs.

### 3.2. The Mutation Rates of L1 5′ UTRs Compared with Those of Other L1 Components

After an L1 element inserts into the human genome, mutations accumulate at different rates at each nucleotide position within the L1 element. These mutation rates are usually measured in substitutions (fixed mutations) per base pair per generation. A full-length human L1 is divided into four components: the 5′ UTR, ORF1, ORF2, and the 3′ UTR. Using the NETWORK 4.6 (http://www.fluxus-engineering.com/sharenet.htm) software [29], we estimated the ages of the six L1 subfamilies, based on the divergence among the 5′ UTRs of all the copies of each subfamily [23, 29]. In addition, we calculated their ages based on the divergence among their 3′ UTRs using the same method. As can be seen in Table 2, the estimated ages of the 443 human-specific L1s averaged 9.90 myrs old, which was calculated based on the divergence among the 5′ UTRs. This estimate is older than 7.75 myrs old, which was calculated based on the divergence among the 3′ UTRs. Previous studies of L1 elements constructed the phylogenetic relationships among different L1 subfamilies using the combined sequences of a partial ORF2 (pORF2) and the 3′ UTR, as most L1 insertions are truncated. It was assumed that L1 elements accumulate mutations at the neutral rate after their insertion [33, 34]. The age estimates of L1 subfamilies based on the divergence among the combined L1 sequences did indeed show that mutations have occurred within human-specific L1s at the neutral rate after their insertion into the host genome [23]. However, as can be seen in Table 2, the mutation rate of the L1 5′ UTR is faster than the neutral rate and faster even than that of the 3′ UTR. This high mutation rate is likely detrimental for many L1 copies in terms of retrotransposition activity, but it could also offer a benefit to L1s if it increases the speed at which these elements can adapt to changing genomic circumstances.

**Figure 2:**
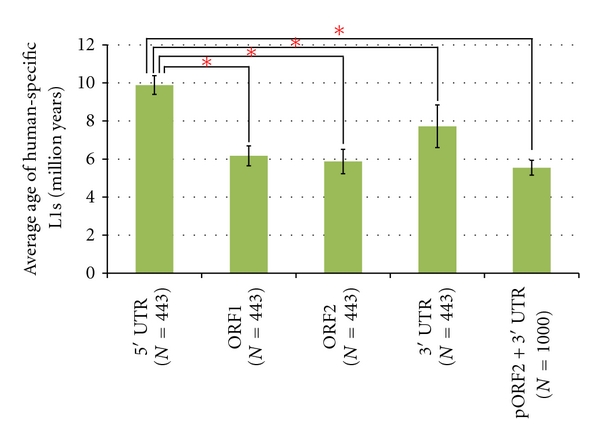
Average age estimates of human-specific L1 elements. *N* represents the number of samples. The pORF2 + 3′ UTR data are from Lee et al. [23]. * indicates significant differences of *P* < 0.0001 in Welch's *t*-test.

We extended this analysis to ORF1 and ORF2 to compare their mutation rates with that of the 5′ UTR. The estimated ages of the 443 human-specific L1s averaged 6.17 and 5.86 myrs old, which were calculated based on divergences among ORF1s and ORF2s of all the copies of each L1 subfamily, respectively. These two age estimates are similar to the estimate of 5.54 myrs old, which was calculated based on the divergence among the combined sequences of the L1s. As shown in Figure 2, we compared the age estimates calculated for each of the four components of the L1s (the 5′ UTR, ORF1, ORF2, and the 3′ UTR) with the age estimate calculated for the combined sequence, using Welch's *t-*test [35]. The standard deviations of the age estimates are nonoverlapping. Especially, the pairwise comparison of the two age estimates based on the divergence among 5′ UTRs and the divergence among 3′ UTRs showed that they are significantly different to one another: the two-tailed *P* value was less than 0.0001. Given these findings, we can assert that the 5′ UTRs of L1s accumulate mutations at a rate faster than the neutral rate. Interestingly, despite this faster mutation rate within the 5′ UTRs, human-specific L1s have shared the same type of L1 5′ UTR since the divergence of humans and chimpanzees [23]. It has been suggested that L1s require host factor(s) for their replication, and that competition between different L1 subfamilies for these limiting resources may have led to the single L1 lineage observed in the human genome today [22]. Thus, it is possible that novel L1 5′ UTRs periodically emerge in the human genome via mutation, but they are not detected in this study as an L1 having a novel 5′ UTR is rapidly lost due to competition with preexisting L1s in the human genome.

### 3.3. The Comparison of Human-Specific L1 5′ UTRs with Chimpanzee-Specific L1 5′ UTRs

The structure of human-specific L1s is the same as that of chimpanzee-specific L1s, although the human-specific L1s have evolved in a single lineage while chimpanzee-specific L1s have evolved in two distinct lineages since the divergence of humans and chimpanzees [23]. Only 19 full-length chimpanzee-specific L1s have been detected in the chimpanzee genome [23]. We recovered these and examined the mutation rate of each component of the chimpanzee-specific insertions. Like their human-specific counterparts, the 5′ UTRs of the chimpanzee-specific elements showed the highest mutation rate (see Supplemetary Table 2). The overall mutation rate of the chimpanzee-specific L1s was by far higher than that of the human-specific L1s, which may have resulted from the relatively small sample size of chimpanzee-specific L1s available for analysis; unlike with the human-specific L1s, the chimpanzee-specific sample size was too small to divide into L1 subfamilies. The necessity of treating the chimpanzee-specific elements, which were likely comprised of members from several different subfamilies, as a single subfamily undoubtedly led to a higher divergence estimate among them and, subsequently, resulted in the high mutation rate of the chimpanzee-specific L1s. On the other hand, the finding that both groups of L1s show a higher mutation rate within their 5′ UTRs relative to the other L1 components or the L1 sequences as a whole is consistent with our other findings.

### 3.4. The Analysis of CpG Dinucleotides within Human-Specific L1 Elements

**Figure 3:**
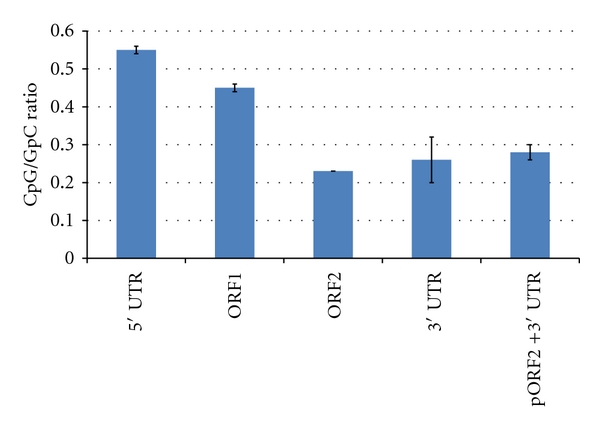
CpG-to-GpC ratio in L1 components. The vertical axis represents the ratio of CpG to GpC dinucleotide loci in each L1 component. The highest ratio is observed in the 5′ UTR.

CpG dinucleotides occur at less than 10% of their expected frequency in the human genome because substitution at a CpG site, caused primarily by the methylation of cytosine, occurs 10- to 50-fold more frequently than other substitutions in primate genomes [36–38]. In spite of this high mutation rate from 5-methyl-CpG to TpG, many cellular functions such as gene expression rely on these CpG dinucleotides [39]. Any genomic region which contains a relatively high density of CpG sites is called a CpG island, and these islands are defined as regions of DNA of at least 200 bp with a GC percentage of greater than 50% and with an observed/expected CpG ratio of greater than 60% [40]. Under these criteria, we searched our L1 component sequences for CpG islands using the CpG island searcher utility (http://cpgislands.usc.edu) [41]. Among the four L1 components, only the 5′ UTR contains a CpG island. To determine whether CpG content is a major factor determining the mutation rates of the L1 components, we compared CpG contents among them. As shown in Figure 3, the 5′ UTR contains the highest density of CpG dinucleotides while ORF2 contains the lowest density. This observation of CpG content mirrored our observation of estimated mutation rates across L1 components, and we therefore wondered whether CpG content may be a major factor influencing the mutation rates of the L1 components. To investigate this hypothesis, we analyzed the correlation between the CpG contents and mutation rates of the L1 components and found that the mutation rate is not correlated with the CpG content (*r* = 0.6665, *P* = 0.5356). Thus, we can state that the relatively high frequency of CpG sites within the L1 5′ UTR is not a major source of the higher mutation rates observed in this component.

## 4. Conclusions

In conclusion, the mutation rate of the L1 5′ UTR is higher than that of the other L1 components, ORF1, ORF2, and the 3′ UTR. However, the TF binding sites are relatively conserved among the human-specific L1 elements. We suspect that any host defense system(s) against L1 elements may be the cause of the higher mutation rate of the 5′ UTR and, in contrast, that L1 elements have kept the TF binding sites conserved against the host defense system(s) in order to survive; it could be the result of the evolutionary battle between L1s and their host. We believe that the increased frequency of substitutions within the 5′ UTRs could play a key role in regulating L1 retrotransposition activity and the number of retrotransposition-competent L1s in the human genome. However, not much is known about the factors causing the high level of sequence diversity in the L1 5′ UTRs. Although we suggest that the relationship between the L1 5′ UTR and other host factors, including the defense systems, causes the high mutation rate of the L1 5′ UTR, we could not rule out other possible factors such as the low fidelity of L1 reverse transcriptase. Thus, more research is needed to explore this intriguing possibility. Finding the factors that cause the increased mutation rates observed within L1 5′ UTRs will shed light on our understanding about L1 evolution and how the human genome may place controls on the retrotransposition rate of its resident L1 element population.

## Supplementary Material

Supplementary Table 1: The genomic information of 443 full-length L1 elements in the human genome was summarized.
Supplementary Table 2: Age of chimpanzee-specific L1 elements was estimated by using the NETWORK 4.6 (http://www.fluxus-engineering.com/sharenet.htm) software. For this calculation, we used 0.15% per site per myr as the nucleotide mutation rate.

## Figures and Tables

**Table 1:** Average frequency of substitutions across the promoter and TF binding sites of the L1 5′ UTR.

Region	Active L1 elements (%)	Dead L1 elements (%)	All L1 elements in the human genome (%)
Sense promoter	0.66	0.99	2.14
YY1	0.15	0.27	1.38
Runx3	0.00	0.26	0.53
SRY-1	0.00	0.49	0.81
Runx3 ASP	0.29	0.26	0.86
SRY-2	0.00	0.00	0.41
5′ UTR overall	0.53	0.78	1.62

**Table 2:** Age estimation of human-specific L1 elements based on each L1 component.

Subfamily^a^	Subfamily^b^	No. of full-length L1 elements	5′ UTR Age ± SD (myrs)	ORF1 Age ± SD (myrs)	ORF2 Age ± SD (myrs)	3′ UTR only Age ± SD (mys)	pORF2 and 3′ UTR Age ± SD (myrs)^b^
L1PA3	L1PA3-1A L1PA3-1Aa L1PA3-1B L1PA3-1Ba L1PA3-1Bb L1PA2-1A	106	20.30 ± 0.65	12.83 ± 0.71	11.31 ± 0.73	15.32 ± 2.11	12.71 ± 0.63

L1PA2	L1PA2-1B L1PA2-1C L1PA2-1D L1PA2-1Da L1PA2-1Db L1PA2-1E	147	13.85 ± 0.38	9.15 ± 0.59	9.63 ± 0.69	11.32 ± 1.14	7.62 ± 0.47

L1HS-1AB	L1HS-1A L1HS-1B	32	8.74 ± 0.53	5.50 ± 0.57	4.88 ± 0.63	10.48 ± 1.87	5.09 ± 0.56

L1HS-preTa	L1HS-preTa	62	7.22 ± 0.41	3.95 ± 0.41	3.79 ± 0.89	4.15 ± 0.84	3.13 ± 0.25

L1HS-Ta0	L1HS-Ta0	38	4.90 ± 0.33	3.25 ± 0.42	3.29 ± 0.47	2.96 ± 0.51	2.73 ± 0.22

L1HS-Ta1	L1HS-Ta1	58	4.38 ± 0.65	2.35 ± 0.43	2.28 ± 0.44	2.29 ± 0.41	1.94 ± 0.20

Average		443^c^	9.90 ± 0.49	6.17 ± 0.52	5.86 ± 0.64	7.75 ± 1.15	5.54 ± 0.39

^a^In this study.

^b^Source Lee et al. [23].

^c^Total number of L1 elements.
