# Supplementary material for: High Levels of Sequence Diversity in the 5′ UTRs of Human-Specific L1 Elements
Source: Comp Funct Genomics. 2012 Feb 7;2012:129416. doi: 10.1155/2012/129416 (PMC3286893; doi:10.1155/2012/129416)
Supplement: Supplementary file 2 [file 129416.f2.docx]

**Supplementary table 2. Age estimation of chimpanzee-specific L1 elements based on each L1 component.**

| **No. of full-length L1 elements** | **5’ UTR Age ± SD**  **(myrs)** | **ORF1 Age ± SD**  **(myrs)** | **ORF2 Age ± SD**  **(myrs)** | **3’ UTR only Age ± SD**  **(mys)** |
| --- | --- | --- | --- | --- |
|  |  |  |  |  |
| **19** | **19.70 ± 1.3** | **11.70 ± 1.3** | **10.13 ± 1.3** | **17.10 ± 2.4** |
